# Accurate *in vivo* tumor detection using plasmonic-enhanced shifted-excitation Raman difference spectroscopy (SERDS)

**DOI:** 10.7150/thno.53101

**Published:** 2021-02-19

**Authors:** Pietro Strobbia, Vanessa Cupil-Garcia, Bridget M. Crawford, Andrew M. Fales, T. Joshua Pfefer, Yang Liu, Martin Maiwald, Bernd Sumpf, Tuan Vo-Dinh

**Affiliations:** 1 Fitzpatrick Institute for Photonics, Duke University, Durham, NC, USA; 2 Department of Biomedical Engineering, Duke University, Durham, NC, USA; 3 Department of Chemistry, Duke University, Durham, NC, USA; 4 Center for Devices and Radiological Health, U.S. Food and Drug Administration, Silver Spring, MD, USA; 5 Ferdinand-Braun-Institut, Leibniz-Institut für Höchstfrequenztechnik, Berlin, Germany

## Abstract

For the majority of cancer patients, surgery is the primary method of treatment. In these cases, accurately removing the entire tumor without harming surrounding tissue is critical; however, due to the lack of intraoperative imaging techniques, surgeons rely on visual and physical inspection to identify tumors. Surface-enhanced Raman scattering (SERS) is emerging as a non-invasive optical alternative for intraoperative tumor identification, with high accuracy and stability. However, Raman detection requires dark rooms to work, which is not consistent with surgical settings.

**Methods:** Herein, we used SERS nanoprobes combined with shifted-excitation Raman difference spectroscopy (SERDS) detection, to accurately detect tumors in xenograft murine model.

**Results:** We demonstrate for the first time the use of SERDS for *in vivo* tumor detection in a murine model under ambient light conditions. We compare traditional Raman detection with SERDS, showing that our method can improve sensitivity and accuracy for this task.

**Conclusion:** Our results show that this method can be used to improve the accuracy and robustness of *in vivo* Raman/SERS biomedical application, aiding the process of clinical translation of these technologies.

## Introduction

For the majority of cancer patients, surgery is the primary treatment method and requires accurate characterization of the tumor location and margins by the surgeon. Due to the lack of simple and safe intraoperative imaging techniques, surgeons commonly rely on visual and physical inspection to identify tumors during surgery. The removal of the entire tumor without affecting surrounding tissue is essential in improving treatment outcomes. However, tumor margins are difficult to identify, leading to tumor recurrence or unnecessary removal of healthy tissue, which can negatively impact long-term survival and patient quality of life 1. Final histopathology reports take days and do not permit margin assessment during surgery. Current practice involves negative margins in surgical resection by resecting normal tissue surrounding the tumor, which can lead to large surgical defects. There is a critical need for intraoperative techniques capable of accurate non-invasive and real-time tumor detection that can enhance the ability of surgeons to precisely remove tumors, improving morbidity and mortality of cancer patients.

Established imaging methods to assist surgeons for tumor resection include magnetic resonance imaging (MRI), computed tomography (CT), and positron emission tomography (PET). These imaging techniques are invasive and expensive and are therefore currently used predominantly for neurosurgery at specialized medical centers 2, 3. The shortcomings of these techniques have fueled research into intraoperative optical imaging to address this unmet clinical need. To this end, NIR-fluorescence techniques have been widely explored for their use with contrast agents 2, 4, 5. However, fluorescent contrast agents suffer from multiple disadvantages such as photobleaching and limited depth penetration. Additionally, autofluorescence from biological structures in the tumor area can affect the signal and lead to false positives 6.

Raman scattering can be used for *in vivo* optical imaging as an alternative to fluorescence 7-9. The vibrational nature of this optical phenomenon produces unique spectral signatures with sharp peaks that are easy to identify over the autofluorescence background. A drawback of this technique is the inherently weak Raman cross section. Surface-enhanced Raman scattering (SERS) can be used to overcome this issue by amplifying the Raman signal 10^7^-10^10^-fold, reaching an ideal sensitivity for *in vivo* applications 10-15. SERS nanoparticles are used as contrast agents in tumor detection, with several unique advantages. These nanoparticles do not suffer from photobleaching, can be multiplexed to image different targets and passively accumulate in tumors through the enhanced permeation and retention (EPR) effect due to their nanometric size. Numerous studies have demonstrated the use of SERS-nanoprobes to detect and image tumors, ranging from wide-area/whole-animal bioimaging to use as endoscopy contrast agents and guidance for tumor resection, among other applications 16-22. SERS is regarded as a powerful emerging technique that can be used intraoperatively to overcome the shortcomings of traditional tumor detection methods. As an example, SERS can be used for the detection of specific expressed biomarkers in tumors and can potentially be used to detect tumors through the cranium 23-26. Our laboratory has developed a surfactant-free and biocompatible synthesis of metallic nanostars suitable for *in vivo* applications 27, 28. These nanostars offer a strong SERS signal enhancement on single particles due to the lightning rod effect of its sharp peaks and can be easily tuned to have surface plasmon resonance in the near infrared (NIR) spectral range, which is ideal for optical detection *in vivo* 27. Our group previously demonstrated that these particles accumulate within tumors through the EPR effect and demonstrated strong SERS signal in tumor tissue^29^. We have also demonstrated the use of gold nanostars for *in vivo* photothermal ablation of primary sarcomas in mice and optically modulated delivery of gold nanostars into brain tumor 29, 30. Furthermore, the combined use of gold nanostars-mediated photothermal therapy and immune-checkpoint inhibition was able to achieve complete eradication of primary treated tumors and distant untreated tumors in mice studies 31, 32. Despite the advances in biomedical applications of SERS, these techniques remain constrained to laboratory-based Raman microscope setups or a dark room where spectroscopy measurements are usually performed. These environments are not ideal for surgical operation as it is important that a surgeon has a strong light source illuminating the area of interest. This light source, in addition to the LED light from instrumentation screens, generates a strong background that can lead to inaccurate Raman detection 33. With the development of methods that can eliminate interfering background signals and permits the accurate measure of Raman spectra, SERS application will see an easy translation to clinical settings. It is also important to note that Raman/SERS tumor identification technologies are yet to be translated into the clinics, while NIR-fluorescence is currently used during surgery. Thereby, while the advantages of Raman are clear, Raman-based technologies have not undergone the same level scrutiny of current state-of-the-art technologies.

Current standard background subtraction methods commonly consist of post-processing data. With these methods, Raman peaks are recognized based on expected features, such as sharp variations in signal intensity as a function of frequency. Despite advancements in post-processing, these methods are inherently incapable of recognizing a Raman peak from background peaks. Post-processing methods can also produce artifacts due to other sources of sharp signal fluctuation (e.g., fluorescence fluctuation and fringes). In addition, Raman peaks can have very different features determined by the laser and optical spectrometer used in the setup, making post-processing methods not easily transferable between different applications. To overcome these limitations, alternative methods have been developed to extract Raman signal based on a physical phenomenon rather than on the digital data. Physical methods involve the modulation of the Raman signal in time (i.e., time-gating), polarization and frequency 34-37. A pure Raman spectrum in these methods can be extracted if the modulation frequency is known, cancelling out all other non-modulated sources of signal.

Although physical methods are very robust and accurate, they often require complex instrumentation, such as pulsed or frequency-modulated lasers or polarization-dependent substrates. Shifted-excitation Raman difference spectroscopy (SERDS) has emerged as a powerful technique for Raman signal extraction based on a physical phenomenon 38-42. This physical method involves the use of a unique laser excitation source that provides two slightly shifted emission lines, close to the bandwidth of the Raman peaks under analysis. Spectra resulting from the excitation with these emission lines will only differ in the frequency at which Raman peaks appear, not modifying any background feature. Thereby, the difference between these spectra will only contain Raman information, cancelling any other source of signal. Recently, a SERDS excitation source was developed with spectral distance between the laser lines of < 2 nm, while fitting in a fiber-coupled small device (i.e., 100 x 60 x 40 mm^3^) 43. The size of this instrument combined with the absence of movable parts, such as external gratings for wavelength tuning, allows for the integration of the source in portable Raman instruments for reliable and robust measurements under field conditions 44. We previously demonstrated that this dual-wavelength SERDS laser source can be combined with SERS-tags in *ex vivo* biomedical application and with SERS nanosensors to enable field detection of molecular biomarkers 45, 46. In our SERDS applications, we demonstrated that this method can be used for the rejection of strong and complex illumination sources, such as fluorescent lights in a plant growth chamber and sunlight in the field 44, 46. The advantages provided by SERDS make it a strong candidate for integration into a portable Raman instrument for accurate Raman measurements in the operating room.

Reported herein is the first demonstration of SERDS to detect tumors in murine models *in vivo.* We first tested the SERDS setup on a 3D-printed phantom recently developed at the U.S. Food and Drug Administration (FDA), to simulate the scattering and strong fluorescence background experienced from human skin 47. We then tested the SERDS setup tumor-bearing mice injected with SERS nanoprobes. In these experiments we used the surfactant-free silver coated gold nanostars coated with silica (AuNS@Ag@SiO_2_), previously developed by our group 28. We demonstrate accurate detection of tumor tissue in a cohort of 6 mice. As shown herein, SERDS can be used to accurately extract the Raman/SERS signal in the laboratory and *in vivo* experiments, both purposely performed in conditions simulating those expected in clinical settings. This work demonstrates for the first time the use of SERDS for *in vivo* tumor detection, paving the way for the adoption of SERDS for real-time intraoperative Raman optical imaging. This approach will aid the translation of Raman applications from lab to clinic, giving surgeons a powerful tool for accurate real-time tumor identification.

## Methods

### Materials

Gold(III) chloride trihydrate (HAuCl4·3H2O), L(+)-ascorbic acid (AA), trisodium citrate dihydrate, sodium borohydride (NaBH4), 1.0 N hydrochloric acid solution (HCl), Dulbecco's phosphate buffered saline (PBS), mercaptobenzoic acid (MBA), fetal bovine serum, and 3,3′-diethylthiatricarbocyanine iodide (DTTC) were supplied by Sigma-Aldrich at the highest purity grade available. Silver nitrate (AgNO3, 99.995%) was purchased from Alfa Aesar. Thiol PEG (mPEG-SH, MW 5000) and silane PEG (mPEG-Si(OCH_2_CH_3_)_3_, MW 5000) were obtained from Nanocs. 1 mL disposable syringes were bought from VWR. Ultrapure water (18 MΩ·cm) was used in all solutions and synthesis. MB49 bladder cancer carcinoma cell line (SCC148) was purchased from Millipore Sigma. Indocyanine green (ICG) was procured from Accutome and prepared in water immediately before use. Tetraethyl orthosilicate was obtained from Tokyo Chemical Industries. The ethanol used in the study was from Koptec. Dulbecco's Modified Eagle Medium 1x with 4.5 g/L D-glucose and L-glutamine (11995-065), penicillin/streptomycin (15140-122), 0.25% tyrpsin-EDTA 1x (25200-56), and HEPES 1 M (15630-080) were purchased from Gibco. Isoflurane (NDC 14043-704-06) was procured from Patterson Veterinary. The paraformaldehyde was purchased as an aqueous solution (16%) from Electron Microscopy Sciences and diluted to the required concentration on the day of the experiments.

### Silver Coated Gold Nanostar (AuNS@Ag) Synthesis & Silica Coating

Polycrystalline spheres for the nanostar reaction were synthesized using a modified version of the Turkevich method 48. Briefly, 15 mL of 1% trisodium citrate were added to 100 mL of a 1 mM boiling solution of HAuCl_4_ under vigorous stirring for 15 min. Finally, the solution was allowed to cool to room temperature, filtered through a 0.22 µm nitrocellulose filter unit, and maintained at 4 ˚C. AuNS@Ag were prepared modifying a previously described method 27, 28. We used AuNS@Ag because the silver-coating was observed to offer an improvement of one order of magnitude in SERS signal with respect to a AuNS 28.

For the lab tests, 10 mL of ultrapure water, 10 µL of 1.0 M HCl and 493 µL of 5.08 mM HAuCl_4_ were added to a round bottom flask stirring vigorously. In quick succession the following were added to the solution: 100 µL of citrate capped seeds (OD_520_ = 2.8), 100 µL of AgNO_3_ (3 mM), and 50 µL of ascorbic acid (0.1 M). After 30 sec of stirring, 50 µL of AgNO_3_ (0.1 M) followed by 10 µL NH_4_OH (30%) were added to initiate the silver coating. After 2 h, MBA was added to the nanoparticle solution to a final concentration of 1 mM. The particles were then centrifuged at 6500 rcf and resuspended in water before using the particles in the SERDS experiments. The MBA-nanoprobes were used in all lab tests.

**Figure 1:**
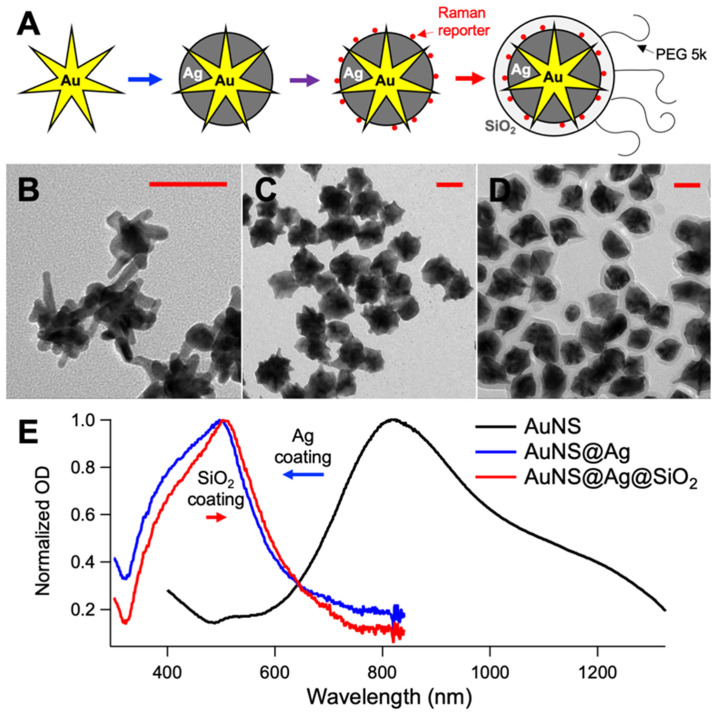
** A.** Schematic representation of the nanoprobes synthesis used in the *in vivo* experiments (intermediates: gold nanostar, silver-coated gold nanostar, coating with DTTC, final nanoprobe). TEM micrographs of the gold nanostars **(B)**, silver-coated gold nanostars **(C)** and final nanoprobes **(D)**. **E.** UV-Vis absorption spectra of the solution during the synthesis. All scale bars are 50 nm.

For the mice injections and *in vivo* test, 400 mL of ultrapure water, 400 µL of 1.0 N HCl and 197 µL of 500 mM HAuCl_4_ were added to a round bottom flask stirring vigorously. In quick succession the following were added to the solution: 8 mL of citrate capped seeds (OD_520_ = 2.8), 400 µL of AgNO_3_ (30 mM), and 500 µL of ascorbic acid (0.4 M). After 30 sec of stirring, 500 µL of AgNO_3_ (0.4 M) followed by 400 µL NH_4_OH (30%) were added to initiate the silver coating. After 2 h, DTTC was added to the nanoparticle solution to a final concentration of 5 µM, and the reaction was allowed to sit overnight. The prepared solution was brought to a final concentration of 1 µM mPEG-SH. Following incubation for 1 hr, the particles were centrifuged at 4000 rcf in 50 mL conical tubes. The particles were dispersed in 2 mL of ultrapure water and 9 mL of ethanol. To coat them with SiO_2,_ 30 µL TEOS 10% and 200 µL NH_4_OH were added to the particle mixture and permitted to incubate for 1 hr. The solution was washed at 4000 rcf and redispersed in 9.5 mL of ethanol and 0.5 mL of ultrapure water. The solution was capped with silane-PEG by adding 50 µL of a 5 mg/mL PEG solution. After incubating particles for 1 h, the particles were washed in ethanol, collected and concentrated 4000x in sterile PBS. Figure 1 shows a schematic representation of the synthesis of the nanoprobes, the TEM micrographs of the intermediates and final products, and the absorption spectra of the solution during the synthesis. For reference, the SERDS extracted spectra of the nanoprobes used in the lab and *in vivo* experiments are reported in Figure S1 of the Supporting Information with the typical Raman bands of the reporters labeled. The DTTC-SiO_2_-coated nanoprobes were used for the studies *in vivo*.

### 3D-printed Tissue Phantom

The 3D-printed phantoms were fabricated based on a previously reported method 47. In brief, the phantoms were designed in SketchUp Free (Trimble) and printed using a mixture of 75%/25% white/clear resin on a Form2 (formlabs), a sterolithography 3D printer that cures liquid photopolymer with UV light. Indocyanine green (λ_fluo_ = 810 nm) was added to the resin to a concentration of 10 nM, to simulate the autofluorescence observed from the skin 49. Fluorescence intensity of ICG in the resin is approximately ten times higher than in water. This printer has a laser spot size of 140 µm with an axial resolution of 25 µm. The phantoms were designed with channels of 1-mm diameter at different depths under the phantom surface, from 0.5 to 3.0 mm. Optical properties of the printer resin were determined using the inverse adding-doubling (IAD) method 50, with transmittance and diffuse reflectance spectra of a 1-mm thick printed sample measured using the 150 mm integrating sphere module of the spectrophotometer (Lambda 1050, PerkinElmer). The absorption and reduced scattering coefficients at 800 nm were of 0.08 and 18.1 cm^-1^, respectively (see Supporting Information Figure S2).

### Cell Culturing Conditions & Animal Model

The MB49 cell lines were maintained at 37 °C in an atmosphere of 5% CO_2_. Complete media for the MB49 consisted of DMEM with 10% fetal bovine serum, 1% penicillin/ streptomycin, and 1% HEPES. The cells were cultured by aspirating culture media and rinsing the flask with warm PBS. After 5-minute incubation with trypsin and cell detachment was observed, 15 mL of complete media was added. Typically, 1 mL of cells (1 x 10^4^ cells) was transferred from previous flask to 25 mL of complete media in a new flask. The cells were subcultured in T-75 flasks and provided with fresh media every 2 to 3 days. Cells were grown to ~ 80% confluency before use and subcutaneously implanted into C57BL/6 mice (250,000 cells) in the right flank. The tumors were permitted to grow for 13 days and then the mice were intravenously (IV) injected through retro orbital injection with the nanoprobes at a dose of 100 µL of 3.3 mg/mL in a sterile PBS solution. The *in vivo* SERDS measurements studies were performed 1 day after the injection of the particles. For the *in vivo* experiments, the mice were anesthetized using isoflurane and laid on heating blanket for the duration of the spectroscopic measurements. The animals were sacrificed on the day of the experiments, their tumors harvested, fixed with 4% paraformaldehyde in PBS overnight, sliced and stained (H&E) by the Animal Pathology Core at the Duke School of Medicine and reviewed by an animal pathologist. All animal studies were approved by the Institutional Animal Care and Use Committee of Duke University (protocol number A176-17-07) and all methods were performed in accordance with guidelines and regulations. Table S1 in the Supporting Information reports the volume of the tumor in each mouse at the time of nanoprobes injection.

### SERDS Optical Setup

A dual-wavelength distributed Bragg reflector (DBR) ridge-waveguide (RW) Y-branch diode laser at 785 nm was used as excitation light source for SERDS. The laser diode is fabricated by metal organic vapor phase epitaxy. The semiconductor chip is described in more detail in a recent report 51. The monolithic device has a footprint of 3 x 0.5 mm^2^ and consists of two laser resonators formed by two DBR gratings on the rear side and the front facet. Ridge waveguides are implemented to guide the laser light and a Y-branch coupler is used to realize a common output aperture. Separate electrical contacts (C_1_, C_2_, C_Y_, and C_out_) allow an individual control of both excitation wavelengths for SERDS. Two on-chip resistor heater elements are implemented close to each DBR grating and enable an adjustable spectral distance between the two excitation lines with respect to the Raman bands under study 52. As described in our previous work, a turn-key laser system was developed at the Ferdinand-Braun-Institut and the dual-wavelength diode laser was integrated into this device 46. The turn-key system provides direct mounting of an optical cage system from outside the housing, which is used for our experiments coupling the laser light via two optical lenses into an optical fiber with a core diameter of 200 µm and a numerical aperture of 0.22. The turn-key system has a compact size of 100 x 60 x 40 mm^3^ and enables integrating the device into portable sensor systems for work in clinical settings. The excitation power used was of 10 mW and 27 mW in the lab and *in vivo* studies, respectively. The excitation wavelengths were adjusted via the on-chip heater elements to 784.1 and 785.5 nm, respectively for λ_0_ and λ_1_.

In lab tests, the excitation fiber was connected to a collimator (Thorlabs) and focused with a 2X microscope objective (NA = 0.1, Thorlabs) on the focal plane of a microscope (Eclipse Ti-U, Nikon). The scattered light is collected using the same optics. Stoke-shifted light passes a notch and a long-pass edge filter (Semrock) and is launched into the collection fiber. In the *in vivo* tests, the SERDS laser is coupled to a RamanProbe (InPhotonics). The laser beam is then focused onto the sample with a working distance of 5 mm. The 180°-backscattered light is collected by the probe. The probes were kept steady on a clamp during the measurements to focus in a specific spot on the anaesthetized mouse. In both setups, the collection fiber is attached to an LS785-PIXIS100 system (Princeton Instruments) with a spectral resolution of 5 cm^-1^. The spectra for lab tests are a sum of 20 accumulations of 10 ms. The spectra for *in vivo* tests are a sum of 25 accumulations of 1 s.

The SERDS reconstruction process was performed by subtracting the λ_0_ raw spectrum from the λ_1_ raw spectrum. This difference spectrum, also called SERDS spectrum, was then flattened subtracting the baseline (polynomial fit of 4th order), to define the 0 in the SERDS spectrum. To remove baseline produced by the integration, the flattened difference spectrum was integrated, smoothed and background-subtracted, using a Savitzky-Golay filter (five-point window and first-order polynomial) on MATLAB, obtaining the integrated SERDS spectrum. This MATLAB Savitzky-Golay filter was also used directly on raw spectra for comparison to generate the background subtracted spectra via post-processing.

The data were also analyzed using principal component analysis (PCA), comparing PCA clustering on raw spectra and SERDS-reconstructed data. PCA was performed on normalized data for both sets on MATLAB, using the singular value decomposition algorithm of the pca.m function of the Statistic and Machine Learning Toolbox.

### Multiphoton microscopy

Multiphoton images were taken using an Olympus FV1000 multiphoton microscope (Olympus America) at the Light Microscopy Core Facility, Duke University. Microscopic imaging was carried out using a femtosecond Ti:Sapphire laser (Chameleon Vision II; Coherent) with tunable wavelength ranging from 680 to 1,080 nm, 140 femtosecond (fsec) pulse width, and 80 MHz repetition rate. The laser beam was focused through a 25× (1.05 NA) water-immersion objective (XLPL25XWMP; Olympus America). Images were taken under 800 nm excitation and 3.7 mW. All images were collected over 10 different z-planes and reconstructed using FIJI (ImageJ).

### UV-Vis and NIR

UV/Vis extinction spectra were acquired with a FLUOstar Omega plate reader (BMG LABTECH). Extinction spectra in the NIR were taken in the Shared Materials Instrumentation Facility, Duke University using a UV-3600 UV-Vis-NIR Spectrophotometer (Shimadzu).

### Transmission electron microscopy

Transmission electron microscopy (TEM) were performed using a Tecnai G2 twin TEM (FEI) at the Shared Materials Instrumentation Facility, Duke University. Nanoparticles were centrifuged, redispersed in water diluted at least 3-fold from the concentration as-synthesized and 10 µL of the solution was drop casted on a TEM grid, formvar/carbon 200 mesh copper (Electron Microscopy Sciences).

## Results and Discussion

### Demonstration of the SERDS signal extraction under clinically relevant light conditions

**Figure 2:**
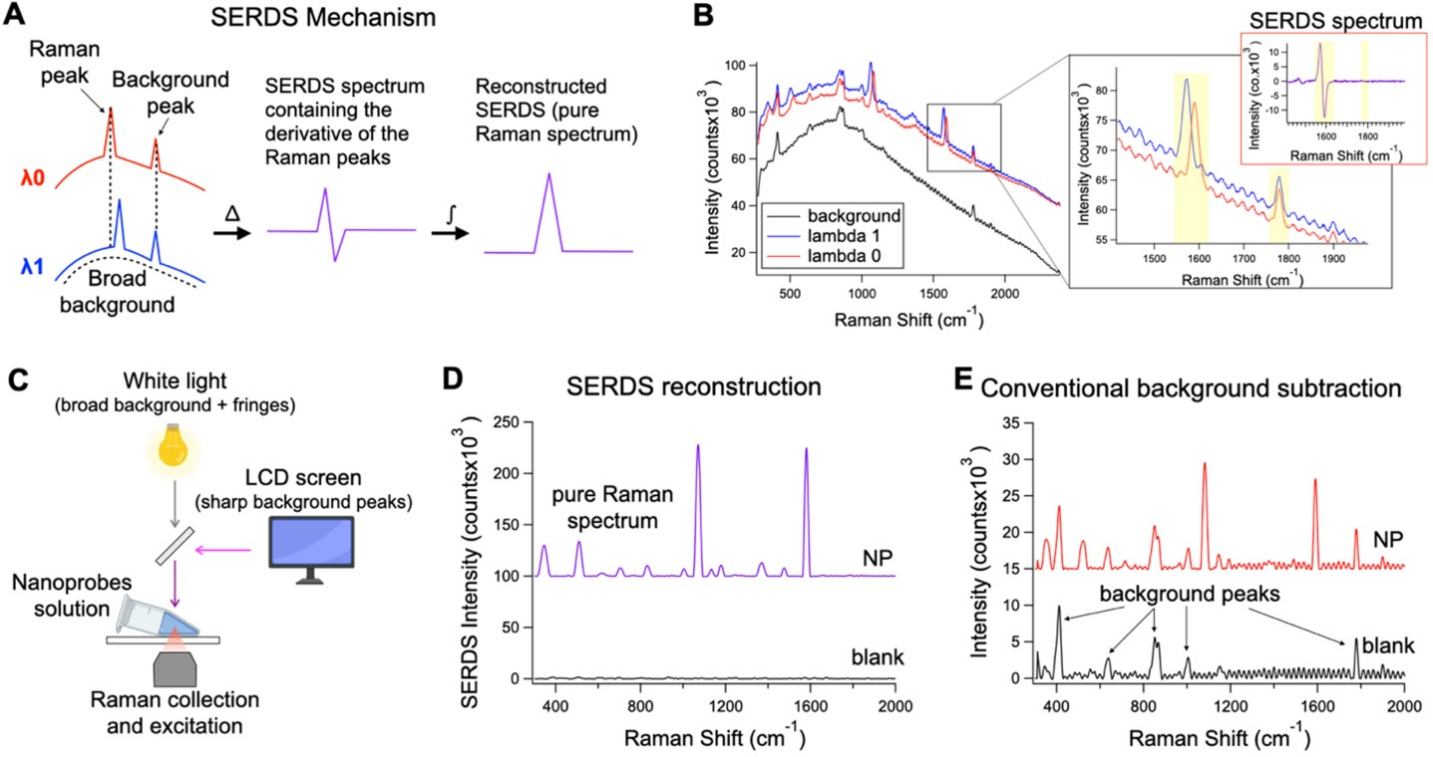
** A.** Theoretical representation of the SERDS mechanism for the extraction of Raman signal in the presence of a complex background signal. **B.** Raw spectra of SERS nanoprobes excited with λ_0_ and λ_1_ and background spectrum. In the inset, a zoom over a region containing a shifted Raman peaks and unmoved background peak from the spectra in B, with the relative SERDS (difference) spectrum of that region. **C.** Schematic representation of the experimental setup used to produce a complex background signal. **D.** Resulting SERDS reconstructed spectra from the spectra in B **E.** Resulting background-subtracted spectra from the spectra in B, containing artifacts and background peaks.

SERDS can be used to perform Raman measurements in complex light settings, giving an accurate reading of the Raman/SERS signal observed. We demonstrated that this feature can be used in SERS applications for tumor identification *in vivo* under conditions expected for intraoperative or clinical settings (Figure 2). Figure 2A describes the basic process to recover the Raman signal via SERDS. Raman spectra are collected using two NIR laser excitations separated by 1.4 nm (i.e., λ_0_ = 784.1 nm and λ_1_ = 785.5 nm), shifting only the Raman peaks as a function of the excitation. The two spectra are subtracted, producing a derivative-like spectrum containing only the Raman information. Integrating the resulting difference spectrum generates a reconstructed SERDS spectrum, which only displays the Raman contribution to the signal. This procedure allows for the elimination of any type of non-Raman background signal, including ambient background light, spectral artifacts and sharp background features. The mechanism of SERDS can be observed in the data shown in Figure 2B, where the spectra for λ_0_ and λ_1_ shift the Raman peaks while keeping constant the background. To demonstrate the efficacy of SERDS in removing complex background signal, we simulated the background that can be experienced in clinical settings and used SERDS to extract the SERS signal in comparison to a conventional form of background subtraction. The background light was simulated by combining a strong broad light source, ubiquitous in clinical and surgical settings, and sharp background peaks, potentially produced in clinical settings by LED indicators and LCD screens (Figure 2C).

Figure 2B shows the shifted Raman spectra of the nanoprobes and the background signal. The latter is composed both of a broad component and of sharp features (background peaks and fringes). The inset in Figure 2B shows the expanded figure of two specific peaks, a Raman band and a background peak from the LCD screen. As it can be observed, only the Raman peak shifts due to the shift of the excitation line using the dual-wavelength diode laser, while the rest of the spectrum remains constant. When the spectra are subtracted (SERDS spectrum), only the Raman information from the spectra are conserved, removing all background sources. The SERDS reconstruction produces a pure Raman spectrum in conventional form that shows the SERS signal from the nanoprobes (NP), as it was demonstrated by performing SERDS reconstruction on a sample not containing nanoprobes (blank) showing no-observable signal (Figure 2D). The NP spectrum shows the typical Raman bands of the reporter used in the nanoprobes. The blank spectrum in Figure 2D has no significant signal because the background signal in this experiment is made of non-Raman contribution and using SERDS we are able to reject all the background signal. To demonstrate the efficacy of SERDS compared to conventional post-processing background subtraction, we used a Savitzky-Golay filter method developed in our laboratory on the same data. This method smooths and flattens the spectrum, highlighting sharp peaks in the data, such as Raman peaks. The results obtained with background subtraction can be observed in Figure 2E. The Savitzky-Golay method is very powerful in highlighting peaks in a spectrum; however, due to its post-processing nature the method cannot distinguish between background peaks, sharp artifacts and Raman peaks, which is true for any post-processing background subtraction. The use of SERDS permit to reject all sharp and broad non-Raman background signal, which cannot be done with post-processing background subtraction. Additionally, when the Raman signal is low with respect to broad background signal, post-processing methods have the potential to erroneously remove Raman peaks and introduce artifacts, due to sharp spectral fluctuations in the noise of the broad background intensity. In contrast, SERDS uses a physical phenomenon (i.e., excitation-dependent wavelength shift) to find and isolate the Raman peaks, which allows it to accurately extract only Raman information.

**Figure 3:**
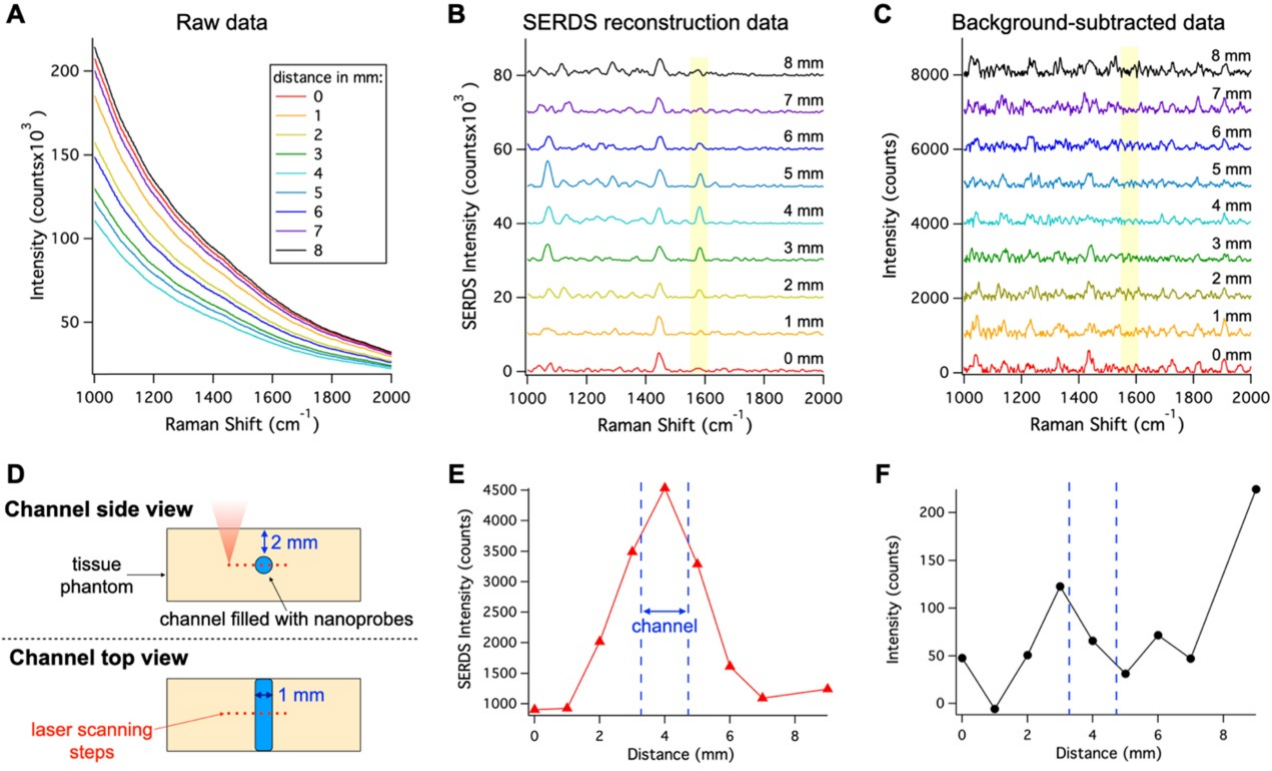
** A.** Raw spectra collected moving the Raman optics laterally across the nanoprobe channel in the 3D-printed phantom with a step of 1 mm. **B.** Resulting SERDS reconstructed spectra from the data in A. **C.** Resulting background-subtracted spectra from the data in A. **D.** Schematic representation of the experiment. Peak intensity for the 1588 cm^-1^ MBA band from the nanoprobes as a function of distance from the SERDS reconstructed **(E)** and from the background-subtracted spectra** (F)**. The spectra in B and C are offset for visualization.

*In vivo* applications of SERS are limited due to the overwhelming background signal caused by autofluorescence, which can cause inaccurate localization of nanoprobes. To show the benefits offered by SERDS in these applications, we demonstrated that SERDS can be used to accurately resolve the Raman signal in a tissue phantom simulating autofluorescence from the skin (Figure 3). The tissue phantom was 3D printed to produce a solid material that matched the reduced scattering and absorption coefficients of tissue, while having NIR fluorescence spectrum of intensity comparable to what has been observed on mouse skin. The phantom contained multiple channels of 1 mm in diameter at different depths. The channel at a 2-mm depth was filled with a nanoprobe solution and scanned with a Raman microscope across its diameter. The resulting raw Raman spectra are reported in Figure 3A. The change in intensity among the spectra is due to a decrease in the fluorescence background when the optical axis of the Raman microscope passes through the channel. As can be observed, the fluorescence signal overwhelms the Raman signal from the nanoprobes, which is not visible in the raw data. Figure 3B shows the SERDS reconstructed spectra where the channel contribution can be observed for the MBA band at 1588 cm^-1^ (highlighted in the figure). By performing a SERDS reconstruction, we are able to extract the Raman signal of the nanoprobes and observe the optical axis crossing the channel at the spectrum corresponding to a 4-mm step. We compare these results with a post processing background subtraction method (Savitzky-Golay filter), shown in Figure 3C. While the SERDS reconstruction is capable of extracting the SERS signal, the post processing method shows inconsistent signal intensities where the MBA bands are expected. The intensities for the 1588 cm^-1^ (highlighted in the figures) band are reported as a function of the displacement of the scanning optics over the nanoprobes channel. Figure 3D is a graphic representation of the scanning experiment and Figure 3E and F reports the SERS intensity results as a function of scanning steps (1 mm/step) across the channel for SERDS reconstruction and background subtracted data, respectively. As can be observed in Figure 3E, the signal from the channel is accurately detected while crossing the channel in the SERDS reconstruction data. The intensity reaches a maximum at 4 mm, when the optical axis in aligned with the channel, and its minima are at 0 and 9 mm, when the focus is furthest away from the channel. In contrast, there is no correlation with the conventionally post-processed data (Figure 3F). Using SERDS reconstruction, we were able to extract only the Raman contribution to the signal and detect the channel over the large background fluorescence, consistent with what is expected in clinical applications of SERS nanoprobes.

We also study how using SERDS can impact the detection of SERS-tags through a highly scattering and fluorescing phantom. The 3D-printed phantom used in this study was fabricated with multiple offset channels at increasing depth below the surface, as previously reported 47. We injected the channels with MBA-nanoprobes and measured focusing the laser at increasing depths. We found that, similarly as for a single depth channel, the SERDS reconstruction outperformed conventional Raman. We observed signal from channels at depth of at least 3.5 mm using the SERDS reconstruction, while for conventional Raman the signal was not observable after 3.0 mm. The results of this study are reported in Figure S3.

### *In vivo* detection of tumors under ambient light conditions

**Figure 4:**
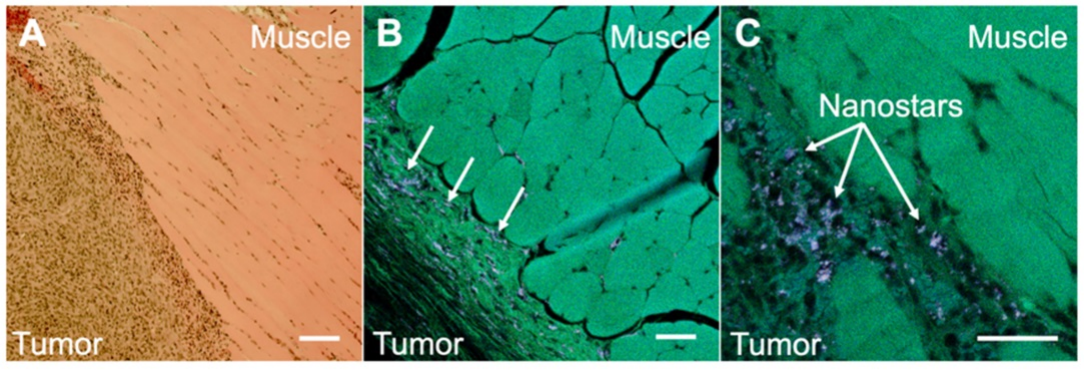
** A.** Histopathology image of a section taken from a harvested flank tumor, showing the tumor boundaries. **B.** Multiphoton image of the same slide as in A, showing the tumor boundaries and the location of the NS (arrows). **C.** Multiphoton image of the same slide as in A, showing the tumor boundaries at a higher magnification. All scale bars are of 100 µm.

To illustrate the potential usefulness of SERDS in clinical applications, mice were injected with bladder carcinoma cells (MB49) in the flank. Tumors were allowed to grow (with tumor sizes ranging from 20 to 102mm^3^) prior to intravenous injection of nanoprobes. Two mice not injected with the nanoprobes were used as negative controls. The presence of the tumors was verified via histopathology. The nanoprobes used in this study used a dye resonant in the NIR as reporter and were coated with a silica shell, to gain more signal enhancement through the resonance Raman effect and to increase stability and biocompatibility, respectively. The nanoprobes' stability was demonstrated by detecting UV-Vis and SERS spectrum of the nanoprobes in water, PBS and in FBS over a time of 24 h (Figure S4 and S5 in the Supporting Information). The stability tests revealed that the particles were stable in buffer and serum (no aggregation observed). A red-shift in the extinction spectrum when incubated in serum was observed, possibly due to protein corona formation. The SERS signal was observed to be unaffected by serum proteins retaining 87% of its original signal after 24 h in serum. Figure 4 shows the comparison between histopathology and multiphoton images of the tumor tissues excised from the mice after the SERS studies. The histopathology images showed a clear distinction between tumor and muscle regions (Figure 4A). When the same tissue was imaged on a multiphoton microscope (Figure 4B and C), we observed the nanostars present in much higher concentration in the tumor tissue than in the muscle. As previously reported, the nanostars appeared as white dots in the multiphoton images due to their strong two-photon luminescence properties 27. The accumulation of nanoprobes, specifically nanostars, in the tumor due to the EPR effect was previously demonstrated by our laboratory 29.

**Figure 5:**
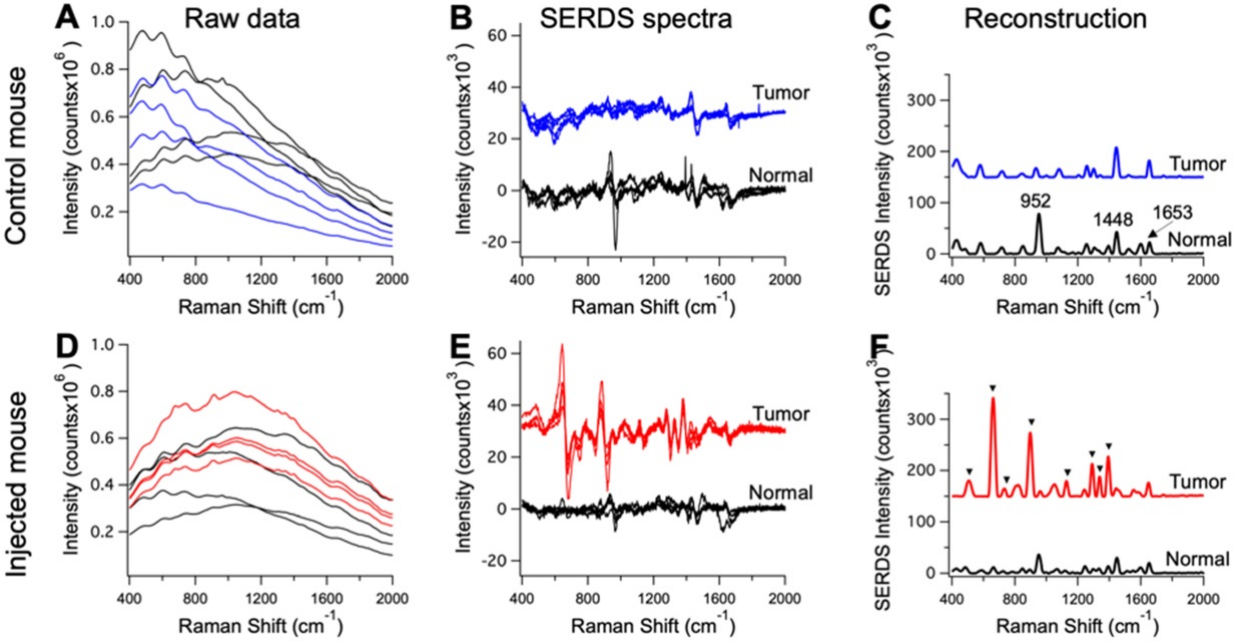
Raw Raman spectra collected on an anesthetized mouse in different points (n = 4) on tumor (blue and red) and normal tissue (black) for a control mouse in **A** and a mouse injected with the nanoprobes in **D**. Resulting SERDS (difference) spectra for each point in A and D, for a control mouse in** B** and a mouse injected with the nanoprobes in **E**. Average reconstructed spectra for tumor and normal tissue in control **(C)** and injected mouse **(F)**. In C the typical Raman bands associated with normal mice tissues are reported. In F, the Raman bands assigned to the Raman reporter on the nanoprobes (i.e., DTTC) are highlighted. The spectra in B, C, E and F are offset for visualization.

The mice injected with nanoprobes, as well as the controls, were used in the *in vivo* SERDS studies. SERDS signals were collected on the anesthetized mice under ambient light using a Raman probe to collect in multiple locations, both on tumors and surrounding normal tissue. Figure 5 displays representative spectra for the measurements performed on the mice. Figure 5A and D shows the raw spectra (n = 4) from the tumor and normal tissue in a control and injected mouse. Even if few peaks are observable, fluorescence signal has the largest contribution in the spectra. The SERDS (n = 4) and average SERDS reconstructed spectra are displayed in Figure 5(B,E) and (C,F), respectively. In Figure 5C, we identified the Raman peaks observed in normal tissue from the mice. A strong peak is observable at 952 cm^-1^ 53. This band is associated to the bone PO_4_^-^ and is due to the fact that one of the spectra was measured on the foot of the mouse, close to the flank on the tumor-side. The other peak at 1448 cm^-1^ is associated with collagen and the CH_2_ bending mode of proteins and lipids 54, 55. The 1653 cm^-1^ is associated to lipids (C=C stretches) and amide (C=O stretching mode of proteins, alpha-helix conformations) 56. In Figure 5F, we identified the peaks associated with the Raman reporter. The full spectrum of the Raman reporter is available in the Supporting Information (Figure S1). These data show the extraction of pure Raman contribution through the SERDS reconstruction in the data collected *in vivo*.

The raw data in Figure 5A and D also show the advantage of using SERS over NIR fluorescence for tumor detection. Our nanoprobes have inherent fluorescence signal given from the Raman reporter used (i.e., DTTC), which fluoresces in the NIR, similar to dyes currently used for intraoperative tumor identification 57. These NIR-fluorescence techniques are based on detection of fluorescence intensity. This detection method can produce inaccurate results due to variation in the intensity of tissue autofluorescence. The results in Figure 5A show the tissue autofluorescence observed in our measurements (no nanoprobes injected). The autofluorescence varied widely, due to the optical setup imaging spots at different light-incidence angles, as well as inherently different autofluorescence. The injected mouse results in Figure 5A show that it is impossible to distinguish between tumor and normal tissue based purely on fluorescence because of the variation in fluorescence intensity due to the autofluorescence background. Using our method, the accuracy of these measurements is improved as we can reject background signal by extracting the pure SERS intensity of the nanoprobes.

**Figure 6:**
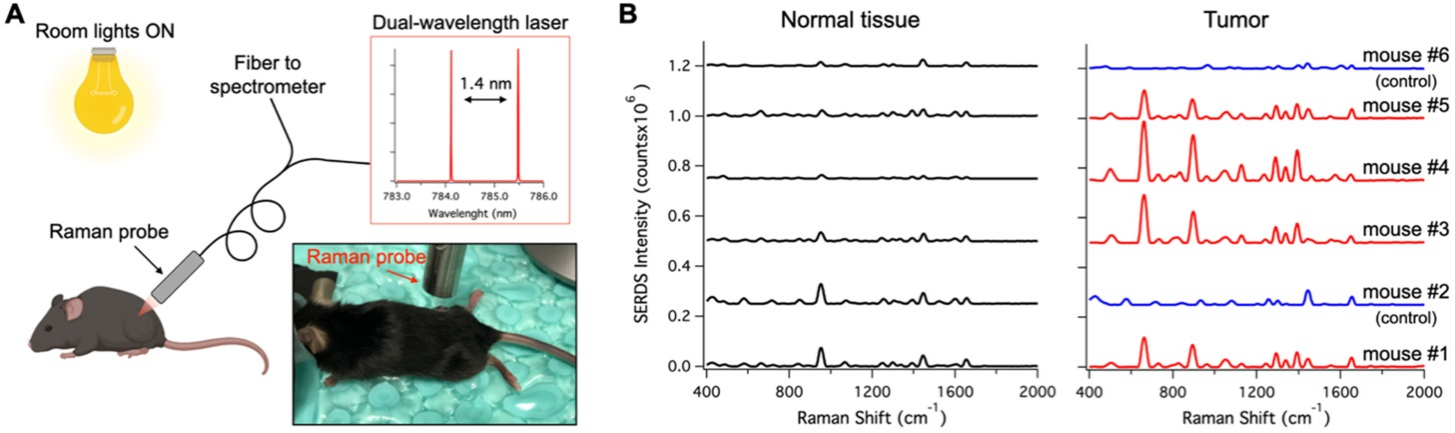
** A.** Schematic and photographic representation of the experimental setup used in the *in vivo* tumor detection studies, using the dual-wavelength SERDS laser, whose emission spectra are reported in the inset. **B.** Average (n = 4 except for Normal tissue - mouse #6 n = 3) SERDS reconstructed spectra of normal tissue and tumor for the different mice studied in this work. The spectra on the right of the panel (Tumor) have the same exact scale as the left (Normal tissue) to permit a visual comparison.

**Figure 7:**
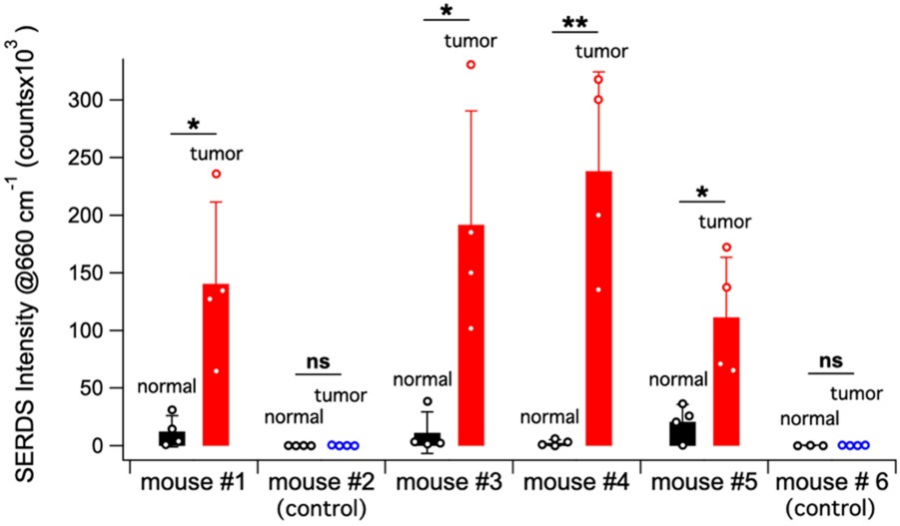
Bar graph reporting the average SERDS intensity of the 663 cm^-1^ Raman reporter band in the for normal tissue and tumor of the different mice in the experiment (control and injected). The intensity of each SERDS measurement is also reported for completeness. The statistical significance for tumor detection (tumor - normal tissue difference) is reported for each mouse.

Figure 6A shows a schematic representation of the *in vivo* studies, within the inset is a photo of the actual setup. The dual-wavelength SERDS laser was coupled into a Raman probe to focus the laser on the mouse skin. Then, the Raman signal was collected through the same probe and detected with a spectrophotometer. The average spectra (n = 4) collected on the tumor or on normal tissue for the 6 mice are reported in Figure 6B. As it can be observed, the SERDS spectra clearly show the Raman bands characteristic of the Raman reporter, in contrast with control and normal tissue. As a further demonstration of tumor detection, the data for the 663-cm^-1^ band of the Raman reporter are reported in the bar graph in Figure 7. The SERDS signals observed for tumors were significantly higher (p < 0.05) than for normal tissue in mice injected with the nanoprobes. The control mice did not show any significant difference between tumor and non-tumor tissues. These studies demonstrate that SERDS can be used to detect tumors with SERS nanoprobes *in vivo* and under ambient light, simulating what is expected in clinical applications.

**Figure 8:**
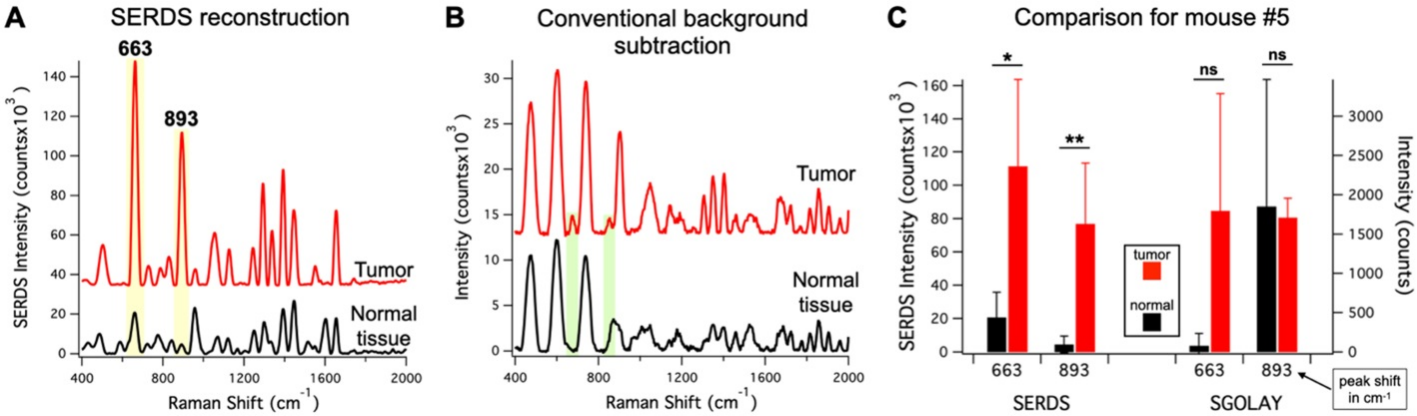
** A.** Average (n = 4) SERDS reconstructed spectra for normal tissue and tumor from mouse #5. **B.** Average (n = 4) background-subtracted spectra for normal tissue and tumor from mouse #5. The major bands of the Raman reporter are highlighted and labeled in the panels. The spectra in A and B are offset for visualization. **C.** Bar graph comparing the average intensity of the 663 and 893 cm^-1^ bands for normal tissue and tumor of mouse #5 for SERDS reconstruction (SERDS) and background-subtracted (SGOLAY) spectra. The statistical significance for tumor detection (tumor - normal tissue difference) is reported for each band in each method.

To demonstrate the advantage of SERDS over post-processing background subtraction in these experimental conditions, we compared our SERDS results with results obtained using conventional background subtraction (Figure 8). We used the data from mouse #5 for the comparison, as this mouse had the smallest intensity difference between tumor and normal tissue. These data are an example of a challenging detection situation that demonstrates how the use of SERDS can be critical. Figure 8A and B show the average SERDS and conventional background-subtracted spectra, respectively. Figure 8C shows the results for the two main Raman reporter bands (highlighted in Figure 8 A and B). The SERDS reconstruction results in significantly different intensities for both bands. In contrast, the conventional background subtraction results showed no significant differences between tumor and normal tissues. As can be seen in the spectra, where in the conventional background subtraction leads to the peaks surrounded by artifact peaks generated by sharp fluctuations in the fluorescence background. When the Raman reporter peaks are small, they are erroneously included in the larger artifact peaks, as it can be clearly seen for the case of the 893-cm^-1^ band. For the 663-cm^-1^ band, the normal tissue intensity for the conventional background subtraction is lower because the method cannot distinguish the peak from the artifacts next to it, which likely causes the large variability in the tumor data. Thereby, the uncertainty generated during post-processing background subtraction due to non-Raman peaks and other forms of background make these methods susceptible to produce inaccurate results. On the other hand, the SERDS procedure allows us to base the extraction of Raman information on a real physical phenomenon, which gives accurate results under ambient light conditions.

To further demonstrated the capabilities of SERDS, we analyzed the *in vivo* measurements using a more complex statistical analysis method (i.e., PCA), applied on the raw set of data and on the SERDS reconstructed data. Figure S6 displays the clustering of the PCA scores of the data for tumor and normal tissue as a function of the first two principal components in the analysis. We found that even with complex statistical analysis methods, the SERDS measurements outperformed conventional Raman spectroscopy. The PCA scores demonstrates that tumor and normal tissue were clearly separable when using SERDS as compared to results from conventional Raman spectroscopy. We believe that these results are due to the fact that, independently of the statistical prediction model used, rejecting any type of interfering signal via SERDS produces more accurate categorization by eliminating spurious variance from the background signal.

## Conclusion

In summary, we demonstrated the benefit offered by the SERDS technique for *in vivo* tumor detection using SERS nanoprobes. We first tested the system with in-laboratory experiments, simulating the conditions expected during surgery in clinical settings. These tests showed that SERDS can be used to achieve greater accuracy than commonly used post-processing background subtraction, due to the ability of specifically recognizing and extracting the Raman signal. Following these results, tumor detection in mice *in vivo* using the SERDS method was demonstrated. SERDS was able to detect tumors in challenging conditions where post-processed spectra did not show significant difference between tumor and normal tissue. The latter method was unable to differentiate between tumor and normal tissue due to the presence of artifacts in the post-processing spectrum, which caused an increase in the signal variability and compromised results accuracy.

In conclusion, the uncertainty generated by artifacts and spurious peaks during post-processing background subtraction make such methods susceptible to reporting inaccurate results. The use of SERDS permits the extraction of Raman information based on a real physical phenomenon, which relay an accurate pure Raman spectrum even under ambient light conditions. In surgical settings, it is crucial that a real-time diagnostic tool is accurate to avoid negatively affecting morbidity and mortality of cancer patients. We herein demonstrate the superior accuracy of our method compared to current standard method for SERS signal extraction under conditions expected in surgical settings (i.e., background lights, strong autofluorescence and *in vivo*). It is noteworthy that the nanoprobes used in this study can work as a theranostics platform for cancer diagnostics as well as for cancer therapy as demonstrated in our previous mouse studies for *in vivo* photothermal treatment of tumors, and photo-immunotherapy to eradicate primary treated tumors and distant untreated tumors and produce an 'anti-cancer vaccine' effect 29, 31, 32. Our method will advance the translation of SERS-based tumor detection to clinical settings by offering a means to minimize inaccurate results in real-time and will permit the use of SERS methods under lighting conditions expected in an operating room.

## Supplementary Material

Supplementary figures and tables.

## References

[1] Mahvi DA, Liu R, Grinstaff MW, Colson YL, Raut CP (2018). Local cancer recurrence: the realities, challenges, and opportunities for new therapies. CA Cancer J Clin.

[2] Vahrmeijer AL, Hutteman M, Van Der Vorst JR, Van De Velde CJ, Frangioni JV (2013). Image-guided cancer surgery using near-infrared fluorescence. Nat Rev Clin Oncol.

[3] Li S, Wu P-H (2013). Magnetic resonance image-guided versus ultrasound-guided high-intensity focused ultrasound in the treatment of breast cancer. Chin J Cancer.

[4] Chan WC, Maxwell DJ, Gao X, Bailey RE, Han M, Nie S (2002). Luminescent quantum dots for multiplexed biological detection and imaging. Curr Opin Biotechnol.

[5] Demétrio de Souza França P, Guru N, Roberts S, Kossatz S, Mason C, Abrahão M (2020). Fluorescence-guided resection of tumors in mouse models of oral cancer. Sci Rep.

[6] Takeuchi Y, Hanaoka N, Hanafusa M, Ishihara R, Higashino K, Iishi H (2011). Autofluorescence imaging of early colorectal cancer. J Biophotonics.

[7] Mahadevan-Jansen A, Richards-Kortum R (1996). Raman spectroscopy for the detection of cancers and precancers. J Biomed Opt.

[8] Jermyn M, Mok K, Mercier J, Desroches J, Pichette J, Saint-Arnaud K (2015). Intraoperative brain cancer detection with Raman spectroscopy in humans. Sci Transl Med.

[9] Bergholt MS, Zheng W, Lin K, Ho KY, Teh M, Yeoh KG (2011). In vivo diagnosis of gastric cancer using Raman endoscopy and ant colony optimization techniques. Int J Cancer.

[10] Jeanmaire DL, Van Duyne RP (1977). Surface raman spectroelectrochemistry: Part I. Heterocyclic, aromatic, and aliphatic amines adsorbed on the anodized silver electrode. J Electroanal Chem Interfacial Electrochem.

[11] Albrecht MG, Creighton JA (1977). Anomalously intense Raman spectra of pyridine at a silver electrode. J Am Chem Soc.

[12] Vo-Dinh T (1998). Surface-enhanced Raman spectroscopy using metallic nanostructures. Trends Analyt Chem.

[13] Sharma B, Frontiera RR, Henry A-I, Ringe E, Van Duyne RP (2012). SERS: Materials, applications, and the future. Mater Today.

[14] Laing S, Jamieson LE, Faulds K, Graham D (2017). Surface-enhanced Raman spectroscopy for in vivo biosensing. Nat Rev Chem.

[15] Strobbia P, Odion RA, Vo-Dinh T (2018). Spectroscopic Chemical Sensing and Imaging: From Plants to Animals and Humans. Chemosensors.

[16] Qian X, Peng X-H, Ansari DO, Yin-Goen Q, Chen GZ, Shin DM (2008). In vivo tumor targeting and spectroscopic detection with surface-enhanced Raman nanoparticle tags. Nat Biotechnol.

[17] Bohndiek SE, Wagadarikar A, Zavaleta CL, Van de Sompel D, Garai E, Jokerst JV (2013). A small animal Raman instrument for rapid, wide-area, spectroscopic imaging. Proc Natl Acad Sci.

[18] Zhang Y, Gu Y, He J, Thackray BD, Ye J (2019). Ultrabright gap-enhanced Raman tags for high-speed bioimaging. Nat Commun.

[19] Garai E, Sensarn S, Zavaleta CL, Loewke NO, Rogalla S, Mandella MJ (2015). A real-time clinical endoscopic system for intraluminal, multiplexed imaging of surface-enhanced Raman scattering nanoparticles. PLoS One.

[20] Karabeber H, Huang R, Iacono P, Samii JM, Pitter K, Holland EC (2014). Guiding Brain Tumor Resection Using Surface-Enhanced Raman Scattering Nanoparticles and a Hand-Held Raman Scanner. ACS Nano.

[21] Gao X, Yue Q, Liu Z, Ke M, Zhou X, Li S (2017). Guiding Brain-Tumor Surgery via Blood-Brain-Barrier-Permeable Gold Nanoprobes with Acid-Triggered MRI/SERRS Signals. Adv Mater.

[22] Zhang Y, Liu Z, Thackray BD, Bao Z, Yin X, Shi F (2018). Intraoperative Raman-Guided Chemo-Photothermal Synergistic Therapy of Advanced Disseminated Ovarian Cancers. Small.

[23] Nayak TR, Andreou C, Oseledchyk A, Marcus WD, Wong HC, Massagué J (2017). Tissue factor-specific ultra-bright SERRS nanostars for Raman detection of pulmonary micrometastases. Nanoscale.

[24] Odion RA, Strobbia P, Crawford BM, Vo-Dinh T (2018). Inverse surface-enhanced spatially offset Raman spectroscopy (SESORS) through a monkey skull. J Raman Spectrosc.

[25] Nicolson F, Andreiuk B, Andreou C, Hsu H-T, Rudder S, Kircher MF (2019). Non-invasive in vivo imaging of cancer using surface-enhanced spatially offset Raman spectroscopy (SESORS). Theranostics.

[26] Ou Y-C, Wen X, Johnson CA, Shae D, Ayala OD, Webb JA (2020). Multimodal Multiplexed Immunoimaging with Nanostars to Detect Multiple Immunomarkers and Monitor Response to Immunotherapies. ACS Nano.

[27] Yuan H, Khoury CG, Hwang H, Wilson CM, Grant GA, Vo-Dinh T (2012). Gold nanostars: surfactant-free synthesis, 3D modelling, and two-photon photoluminescence imaging. Nanotechnology.

[28] Fales AM, Yuan H, Vo-Dinh T (2014). Development of Hybrid Silver-Coated Gold Nanostars for Nonaggregated Surface-Enhanced Raman Scattering. J Phys Chem C.

[29] Liu Y, Ashton JR, Moding EJ, Yuan H, Register JK, Fales AM (2015). A plasmonic gold nanostar theranostic probe for in vivo tumor imaging and photothermal therapy. Theranostics.

[30] Yuan H, Wilson CM, Xia J, Doyle SL, Li S, Fales AM (2014). Plasmonics-enhanced and optically modulated delivery of gold nanostars into brain tumor. Nanoscale.

[31] Liu Y, Maccarini P, Palmer GM, Etienne W, Zhao Y, Lee C-T (2017). Synergistic immuno photothermal nanotherapy (SYMPHONY) for the treatment of unresectable and metastatic cancers. Sci Rep.

[32] Liu Y, Chongsathidkiet P, Crawford BM, Odion R, Dechant CA, Kemeny HR (2019). Plasmonic gold nanostar-mediated photothermal immunotherapy for brain tumor ablation and immunologic memory. Immunotherapy.

[33] Desroches J, Laurence A, Jermyn M, Pinto M, Tremblay M-A, Petrecca K (2017). Raman spectroscopy in microsurgery: impact of operating microscope illumination sources on data quality and tissue classification. Analyst.

[34] Scaffidi JP, Gregas MK, Lauly B, Carter JC, Angel SM, Vo-Dinh T (2010). Trace molecular detection via surface-enhanced Raman scattering and surface-enhanced resonance Raman scattering at a distance of 15 meters. Appl Spectrosc.

[35] Hong KY, Brolo AG (2017). Polarization-dependent surface-enhanced Raman scattering (SERS) from microarrays. Anal Chim Acta.

[36] Strobbia P, Sadler T, Odion RA, Vo-Dinh T (2019). SERS in Plain Sight: A Polarization Modulation Method for Signal Extraction. Anal Chem.

[37] De Luca A, Dholakia K, Mazilu M (2015). Modulated Raman Spectroscopy for Enhanced Cancer Diagnosis at the Cellular Level. Sensors.

[38] Mosier-Boss P, Lieberman S, Newbery R (1995). Fluorescence rejection in Raman spectroscopy by shifted-spectra, edge detection, and FFT filtering techniques. Appl Spectrosc.

[39] Zhao J, Carrabba MM, Allen FS (2002). Automated Fluorescence Rejection Using Shifted Excitation Raman Difference Spectroscopy. Appl Spectrosc.

[40] McCain ST, Willett RM, Brady DJ (2008). Multi-excitation Raman spectroscopy technique for fluorescence rejection. Opt Express.

[41] Maiwald M, Erbert G, Klehr A, Kronfeldt HD, Schmidt H, Sumpf B (2006). Rapid shifted excitation Raman difference spectroscopy with a distributed feedback diode laser emitting at 785 nm. Appl Phys B.

[42] Gebrekidan MT, Knipfer C, Stelzle F, Popp J, Will S, Braeuer A (2016). A shifted-excitation Raman difference spectroscopy (SERDS) evaluation strategy for the efficient isolation of Raman spectra from extreme fluorescence interference. J Raman Spectrosc.

[43] Maiwald M, Eppich B, Fricke J, Ginolas A, Bugge F, Sumpf B (2014). Dual-wavelength Y-branch distributed Bragg reflector diode laser at 785 nanometers for shifted excitation Raman difference spectroscopy. Appl Spectrosc.

[44] Maiwald M, Müller A, Sumpf B, Tränkle G (2016). A portable shifted excitation Raman difference spectroscopy system: device and field demonstration. J Raman Spectrosc.

[45] Register J, Maiwald M, Fales A, Strobbia P, Sumpf B, Vo-Dinh T (2018). Shifted-excitation Raman difference spectroscopy for the detection of SERS-encoded gold nanostar probes. J Raman Spectrosc.

[46] Strobbia P, Odion RA, Maiwald M, Sumpf B, Vo-Dinh T (2020). Direct SERDS sensing of molecular biomarkers in plants under field conditions. Anal Bioanal Chem.

[47] Fales AM, Strobbia P, Vo-Dinh T, Ilev IK, Pfefer TJ (2020). 3D-printed phantoms for characterizing SERS nanoparticle detectability in turbid media. Analyst.

[48] Turkevich J, Stevenson PC, Hillier J (1951). A study of the nucleation and growth processes in the synthesis of colloidal gold. Discuss Faraday Soc.

[49] De Grand AM, Lomnes SJ, Lee DS, Pietrzykowski M, Ohnishi S, Morgan TG (2006). Tissue-like phantoms for near-infrared fluorescence imaging system assessment and the training of surgeons. J Biomed Opt.

[50] Prahl SA, van Gemert MJC, Welch AJ (1993). Determining the optical properties of turbid media by using the adding-doubling method. Appl Opt.

[51] Sumpf B, Maiwald M, Müller A, Fricke J, Ressel P, Bugge F (2015). Comparison of two concepts for dual-wavelength DBR ridge waveguide diode lasers at 785 nm suitable for shifted excitation Raman difference spectroscopy. Appl Phys B.

[52] Maiwald M, Sumpf B, Tränkle G (2018). Rapid and adjustable shifted excitation Raman difference spectroscopy using a dual-wavelength diode laser at 785 nm. J Raman Spectrosc.

[53] Huang N, Short M, Zhao J, Wang H, Lui H, Korbelik M (2011). Full range characterization of the Raman spectra of organs in a murine model. Opt Express.

[54] Stone N, Matousek P (2008). Advanced Transmission Raman Spectroscopy: A Promising Tool for Breast Disease Diagnosis. Cancer Res.

[55] Kaminaka S, Yamazaki H, Ito T, Kohda E, Hamaguchi Ho (2001). Near-infrared Raman spectroscopy of human lung tissues: possibility of molecular-level cancer diagnosis. J Raman Spectrosc.

[56] Shafer-Peltier KE, Haka AS, Motz JT, Fitzmaurice M, Dasari RR, Feld MS (2002). Model-based biological Raman spectral imaging. J Cell Biochem.

[57] Hu Z, Fang C, Li B, Zhang Z, Cao C, Cai M (2020). First-in-human liver-tumour surgery guided by multispectral fluorescence imaging in the visible and near-infrared-I/II windows. Nat Biomed Eng.

