# Supplementary material for: Accurate in vivo tumor detection using plasmonic-enhanced shifted-excitation Raman difference spectroscopy (SERDS)
Source: Theranostics. 2021 Feb 19;11(9):4090–102. doi: 10.7150/thno.53101 (PMC7977455; doi:10.7150/thno.53101)
Supplement: Supplementary file 1 — Supplementary figures and tables. [file thnov11p4090s1.pdf]

SUPPORTING INFORMATION

Accurate *in vivo* tumor detection using plasmonic-enhanced shifted-excitation Raman difference spectroscopy (SERDS)

Pietro Strobbia[1,2], Vanessa Cupil-Garcia[1,3], Bridget M. Crawford[1,2], Andrew M. Fales[4], T. Joshua Pfefer[4], Yang Liu[1,2], Martin Maiwald[5], Bernd Sumpf[5], Tuan Vo-Dinh[1,2,3(*)]

[1] Fitzpatrick Institute for Photonics, Duke University, Durham, NC, USA

[2] Department of Biomedical Engineering, Duke University, Durham, NC, USA

[3] Department of Chemistry, Duke University, Durham, NC, USA

[4] Center for Devices and Radiological Health, U.S. Food and Drug Administration, Silver Spring, MD, USA

[5] Ferdinand-Braun-Institut, Leibniz-Institut für Höchstfrequenztechnik, Berlin, Germany

(*) Corresponding author: tuan.vodinh@duke.edu

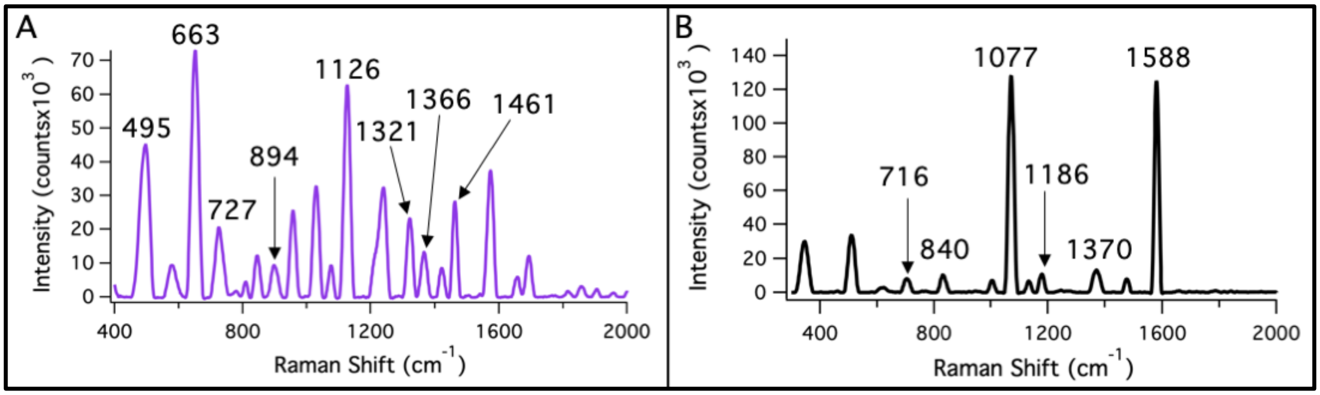

Figure S1. **A**. SERDS spectrum of the nanoprobes solution used in in vivo studies (SiO_2-coated-Ag-coated gold nanostars, Raman reporter = DTTC). **B**. SERDS spectrum of the nanoprobes solution used in in lab tests (Ag-coated gold nanostars, Raman reporter = MBA).

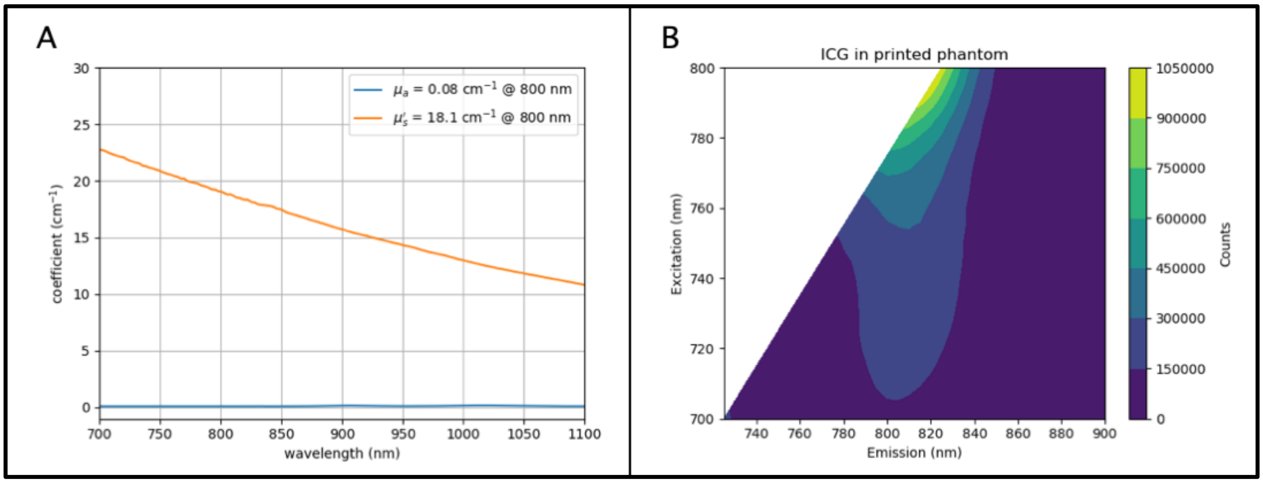

Figure S2. **A**. Scattering and absorption coefficient of the 3D-printed tissue phantoms used in the lab tests as a function of wavelength (μ_a @800 nm = 0.08 cm^{-1}, μ_S @800 nm = 18.1 cm^{-1}). **B**. Excitation-emission matrix for the fluorescence of ICG-3D-printed tissue phantom for excitation in the NIR.

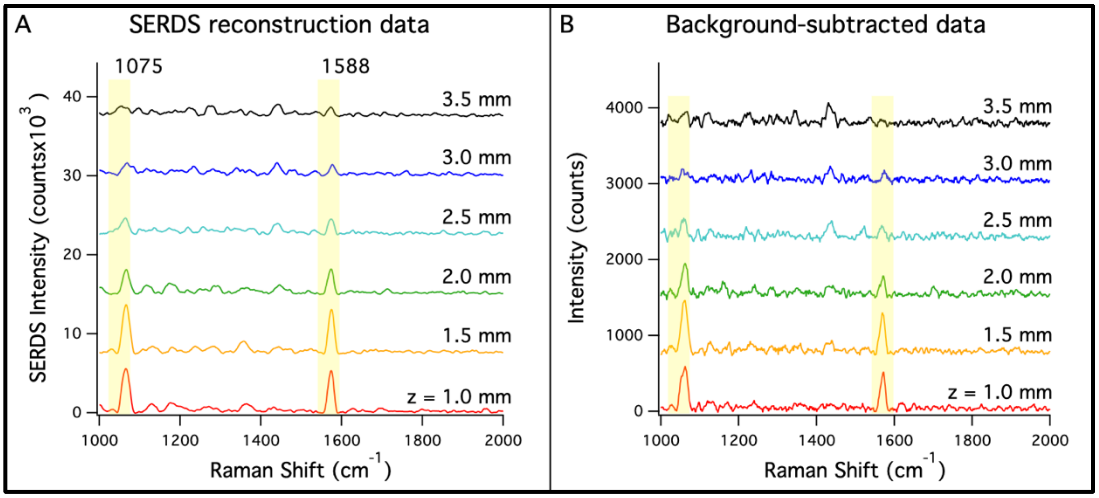

Figure S3. SERDS (**A**) and background subtracted (**B**) spectra measured on a tissue phantom with MBA-nanoprobes injected in channels at different depth. Each spectrum is labeled with the relative channel depth. The two main MBA bands are labeled and highlighted in the spectra.

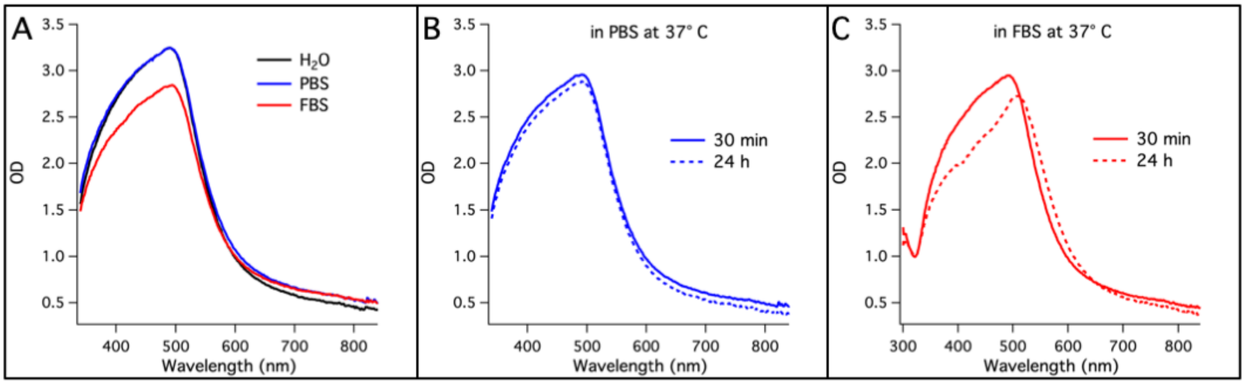

Figure S4. **A**. UV-Vis extinction spectra for the nanoprobes used in the *in vivo* studies in different media: water (H2O), phosphate buffer saline (PBS), 90% fetal bovine serum (FBS). **B**. UV-Vis extinction spectra for the nanoprobes in PBS at different time points. **C**. UV-Vis extinction spectra for the nanoprobes in FBS at different time points.

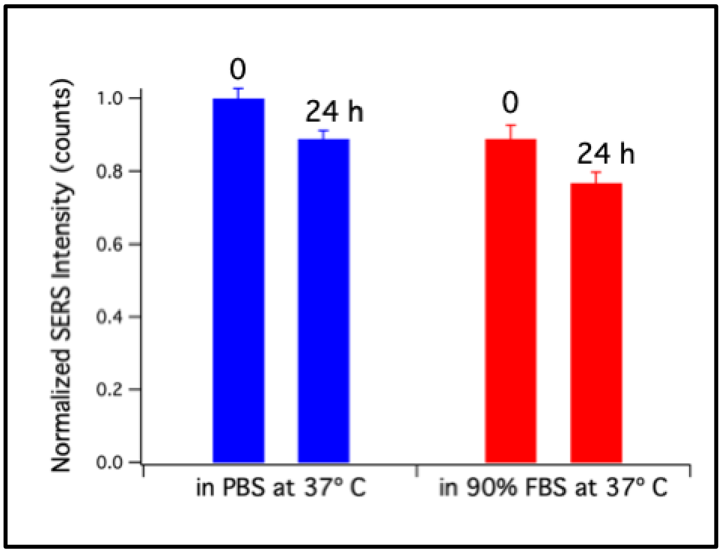

Figure S5. SERS intensities at 663 cm^{-1} for the nanoprobes used in the *in vivo* studies in different media: phosphate buffer saline (PBS), 90% fetal bovine serum (FBS) at different time points.

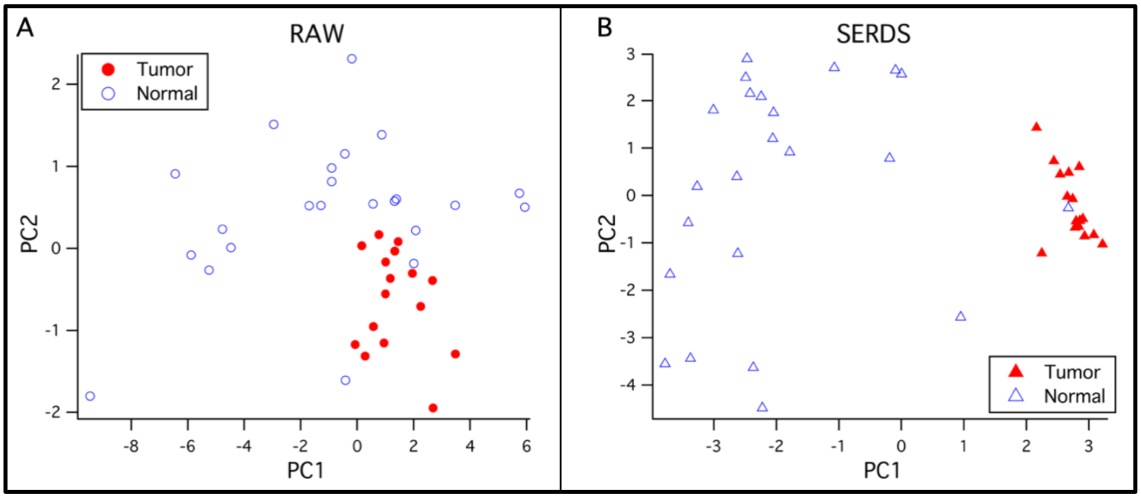

Figure S6. PCA analysis results for the *in vivo* measurements using the raw data set (**A**) and the SERDS reconstructed data (**B**). Reporter here are the PCA scores for the first two principal components for each set with data labeled based on the tissue type. Tumor data for control mice were excluded from this analysis.

Table S1. Tumor volume at the time of SERS-tag injection for the mice studied.

Mouse #	Tumor volume (mm^3)
1	102.1
2 (control)	30.1
3	20.3
4	32.7
5	73.3
6 (control)	55.5
